# Sub-millielectronvolt Line Widths in Polarized Low-Temperature Photoluminescence of 2D PbS Nanoplatelets

**DOI:** 10.1021/acs.nanolett.4c04402

**Published:** 2024-11-22

**Authors:** Pengji Li, Leon Biesterfeld, Lars F. Klepzig, Jingzhong Yang, Huu Thoai Ngo, Ahmed Addad, Tom N. Rakow, Ruolin Guan, Eddy P. Rugeramigabo, Ivan Zaluzhnyy, Frank Schreiber, Louis Biadala, Jannika Lauth, Michael Zopf

**Affiliations:** † Institute of Solid State Physics, Leibniz University Hannover, Appelstraße 2, D-30167 Hannover, Germany; ⊗ Cluster of Excellence PhoenixD (Photonics, Optics, and Engineering − Innovation Across Disciplines), Welfengarten 1A, D-30167 Hannover, Germany; § Institute of Physical and Theoretical Chemistry, Eberhard Karls University of Tübingen, Auf der Morgenstelle 18, D-72076 Tübingen, Germany; ∥ Institute of Physical Chemistry and Electrochemistry, Leibniz University Hannover, Callinstr. 3A, D-30167 Hannover, Germany; ⊥ Université de Lille, CNRS, Centrale Lille, Université Polytechnique Hauts-de-France, Junia-ISEN, UMR 8520 - IEMN, F-59000 Lille, France; # Université Lille, CNRS, INRAE, Centrale Lille, UMR 8207 − UMET- Unité Matériaux et Transformations, F-59000 Lille, France; ∇ Institute of Applied Physics, Eberhard Karls University of Tübingen, Auf der Morgenstelle 10, D-72076 Tübingen, Germany; × Laboratory of Nano and Quantum Engineering, Leibniz University Hannover, Schneiderberg 39, D-30167 Hannover, Germany

**Keywords:** PbS nanoplatelets, cryogenic temperatures, polarized photoluminescence, sub-meV line widths, trions

## Abstract

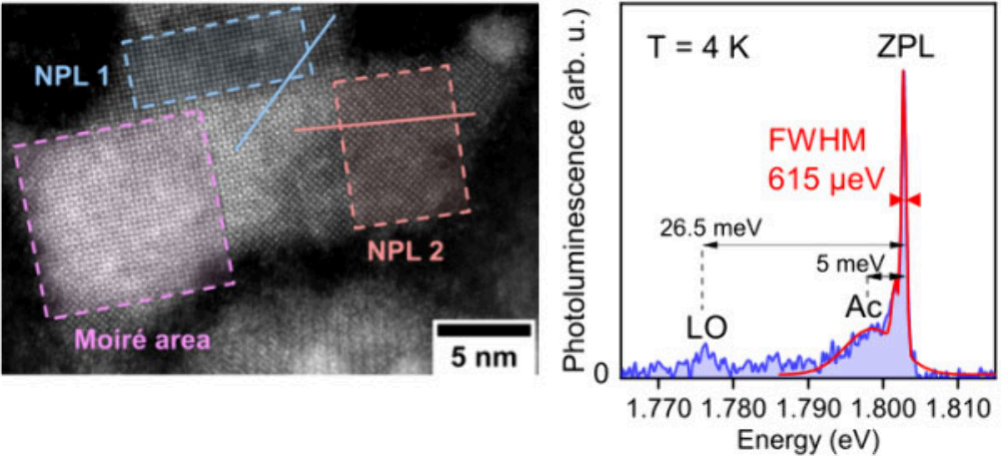

Colloidal semiconductor nanocrystals are promising materials for classical and quantum light sources due to their efficient photoluminescence (PL) and versatile chemistry. While visible emitters are well-established, excellent (near-infrared) sources are still being pursued. We present the first comprehensive analysis of low-temperature PL from two-dimensional (2D) PbS nanoplatelets (NPLs). Ultrathin 2D PbS NPLs exhibit high crystallinity confirmed by scanning transmission electron microscopy, revealing Moiré patterns in overlapping NPLs. At 4 K, unique PL features are observed in single PbS NPLs, including narrow zero-phonon lines with line widths down to 0.6 meV and a linear degree of polarization up to 90%. Time-resolved measurements identify trions as the dominant emission source with a 2.3 ns decay time. Sub-meV spectral diffusion and no inherent blinking over minutes are observed, as well as discrete spectral jumps without memory effects. These findings advance the understanding and underscore the potential of colloidal PbS NPLs for optical and quantum technologies.

Colloidal semiconductor nanocrystals are being extensively studied for their use as classical and quantum light sources due to their optical properties dominated by size quantization.^1−3^ Key requirements for their application include photostable PL with high quantum yields, short radiative lifetimes, low spectral broadening and diffusion, as well as scalable fabrication. At UV to visible wavelengths, cadmium chalcogenide CdX (X = S, Se, and Te) NPLs and heterostructures are known for their excellent optical properties. In particular, CdSe-based NPL systems exhibit narrow line widths below 40 meV at room temperature,^4,5^ between 80% and up to unity PL quantum yield,^6−8^ fast radiative decay in the nanosecond range,^4,9^ and highly directional PL.^10^ In recent years, colloidal lead halide perovskite nanocrystals have emerged as emitters with efficient (quantum yield over 96%),^11^ narrow (12–42 nm) and rapidly decaying (1–29 ns) room-temperature PL at visible wavelengths,^12^ as well as single photon emission.^13−15^ However, materials with similar characteristics at (near-)infrared (NIR) wavelengths are yet highly sought for, in particular for photonic quantum communication applications.^16−18^ Potential candidates include Ag-doped CdSe NPLs,^19^ HgTe NPLs^20^ or InAs/CdSe core–shell nanocrystals.^21^ Another promising material class are lead chalcogenide PbX (X = S, Se, Te) QDs,^22−24^ NPLs^25−27^ and related heterostructures:^28−30^ For instance, Krishnamurthy et al. demonstrated single spherical PbS/CdS QDs emitting in the telecom O-band (near 0.95 eV) at room-temperature, featuring photon antibunching and an average line width of 89.5 meV.^29^ In a similar system and at *T* = 4 K, Hu et al. reported on bleaching-free PL at around 1.0 eV and mean intrinsic PL line width of 16.4 meV, featuring asymmetric line shapes caused by the coupling of excitons to optical and acoustic phonon modes.^28^ In both cases, the broad line widths (compared to their II–VI analogues, such as CdSe QDs) are a result of a 64-fold degenerated band-edge exciton in PbX QDs that splits into multiple energetically similar transitions, resulting in intrinsic PL broadening.^28,31,32^ A closely related, yet unexplored system at the single particle level are photoluminescent 2D PbS NPLs. Manteiga Vázquez et al. developed a synthesis of rock salt cubic-structured PbS NPLs exhibiting a PL quantum yield of up to 19.4% for PL at 1.7 eV (720 nm) upon surface passivation with CdCl_2_.^25^ This strongly enhanced emission efficiency provides the opportunity to investigate the excitonic emission properties of 2D PbS NPLs at the individual emitter level and exploring their electronic structure, phonon interactions and spectral characteristics at cryogenic temperatures.

Our findings provide the first in-depth optical study of individual PbS NPLs at cryogenic temperatures. Highly polarized emissions at around 1.82 eV (677.6 nm) with sub-meV line widths are observed at *T* = 4 K, accompanied by an acoustic phonon sideband. Time-resolved and excitation power dependent measurements reveal trion states as the dominant cause of PL. The emissions exhibit exceptional spectral stability with sub-meV spectral diffusion and are blinking-free over several minutes, promoting the potential of 2D PbS NPLs for reaching near-infrared optoelectronic applications.

NIR emitting colloidal PbS NPLs passivated with CdCl_2_ were synthesized by a method described by Manteiga Vázquez et al.^25^Figure 1a shows an overview TEM image of PbS NPLs resembling a rectangular shape with average lateral dimensions of (16.0 ± 1.6) × (9.2 ± 1.2) nm^2^ and a corresponding aspect ratio of 1.7:1 (see Figure S1 for an additional overview image and the corresponding size histogram). Figure 1b depicts a HR-HAADF-STEM image of two overlapping PbS NPLs (the corresponding FFT patterns of the highlighted crystal areas are shown in Figure S2a). The individual PbS NPLs are highly crystalline and exhibit the cubic rock salt structure (space group Fm3̅m) expected for 2D PbS nanosheets (NSs) and NPLs^25,33,34^ with the characteristic lattice spacings of 2.9 Å (200) and 2.1 Å (220) (PDF card 01-078-1900). Notably, no diffraction peaks of an orthorhombic PbS phase (interplane distances of 2.8 and 2.05 Å)^35^ are evident from the FFT patterns (see Figure S2). The ultrathin 2D geometry and crystalline nature of the NPLs is underpinned by the formation of a pronounced Moiré pattern in the overlapping area of the two differently oriented diffracting crystals (see Figure S2b, c for additional examples). Although not directly related to the in-depth optical studies in this work, the formation of randomly oriented Moiré patterns suggests that the small CdCl_2_ ligands used as X- and Z-type ligands in a postsynthetic surface passivation step, allow for a quasi-direct contact between some PbS NPLs (in contrast to typical bulky organic surfactants such as oleic acid, which lead to further spatially separated NPLs, see also Figure S3 for grazing-incidence wide-angle X-ray scattering data of PbS NPLs and further discussion).^26,36^ While twistronics are very thoroughly researched for van der Waals materials,^37^ Moiré superlattices based on metavalently bound materials such as PbS have only recently been accessed by Wang et al. via an aqueous synthesis route with readily removable ligands. We assume that metal halide passivation can yield similar formations while at the same time enhancing the optical properties of the PbS NPLs synthesized in organic medium.^36^Figure 1c depicts the optical characteristics of the PbS NPL ensemble in colloidal solution at room-temperature, which exhibit an excitonic absorption feature at 1.96 eV and associated NIR PL at 1.70 eV with a rather broad fwhm of 264 meV (Figure 1c). To gain further insight into the optical, structural and electronic properties of PbS NPLs at the single NPL level, we perform PL measurements at cryogenic temperatures (see SI, section A. Please note that signs of Moiré modulation are not expected in the characterized single NPL optical spectra since the NPLs are separated spatially by the polystyrene matrix).

**Figure 1:**
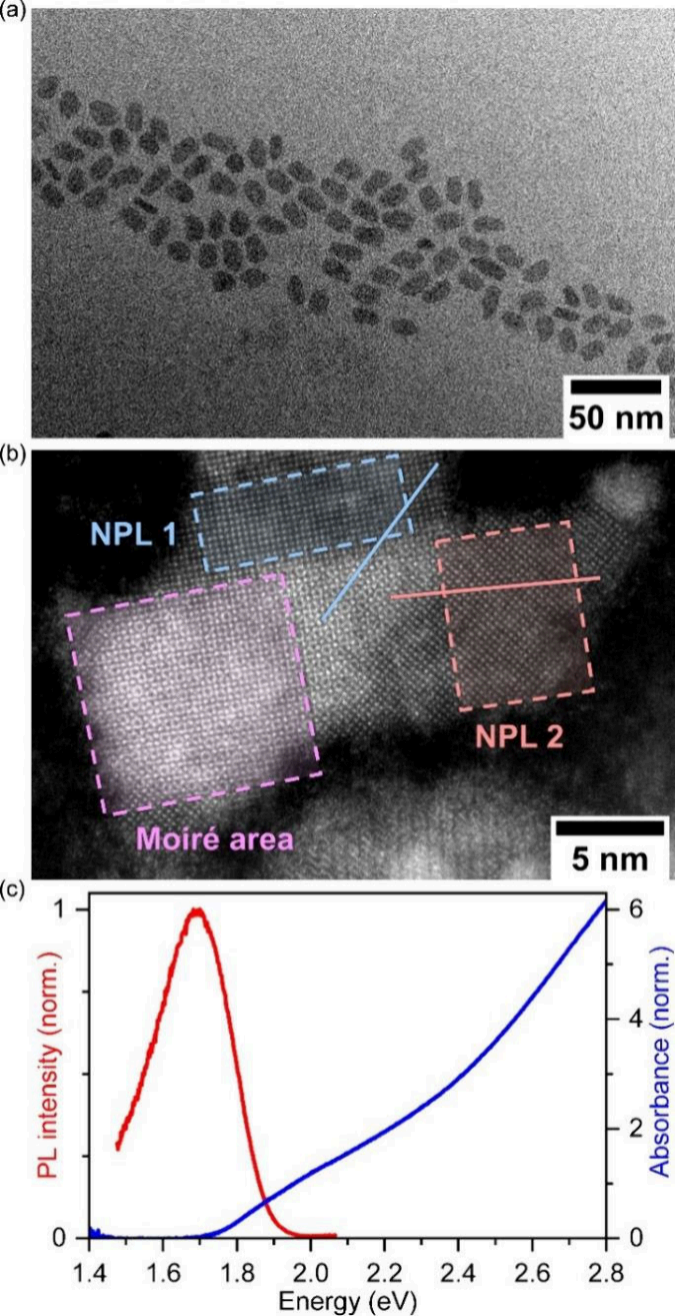
(a) Overview TEM image of rectangular PbS NPLs exhibiting average lateral dimensions of (16.0 ± 1.6) × (9.2 ± 1.2) nm^2^. (b) HR-HAADF-STEM image of two overlapping PbS NPLs forming a Moiré pattern. The formation of the interference pattern emphasizes the ultrathin 2D geometry of the PbS NPLs. (c) Ensemble room temperature absorbance and PL spectrum of PbS NPLs (in colloidal solution), exhibiting excitonic absorption at 1.96 eV and NIR PL at 1.70 eV.

Figure 2a shows a representative PL spectrum of individual PbS NPLs at *T* = 4 K. In marked contrast with typical PL spectra of PbS nanocrystals for which single broad (≥8 meV) emission lines are observed,^28^ the PL spectra of PbS NPLs consist of a narrow, resolution-limited, zero phonon line (ZPL) together with phonon sidebands at energies of 26.5 and 5 meV that we attribute to optical (LO) and acoustic phonons (Ac), respectively^28,38^ (see Table S1 for the fitting results, Figures S5 and S6 for more spectra of individual NPLs). The statistics on more than 70 individual NPLs show that the emission energies of the ZPL (Figures 2b) is centered around 1.82 ± 0.06 eV, which clearly indicates a high level of uniformity in the thickness and the lateral dimensions of the PbS NPLs. Moreover, Figure 2c shows that the NPL emission line width does not exceed 2 meV and that more than 74% of the studied PbS NPLs exhibit a sub-meV line width. The detected line widths go down to 0.6 meV, approaching the resolution limit of the spectrometer. This strongly contrasts with the sharpest emission line width measured on individual PbS nanocrystals (≥8 meV)^28^ for which the line broadening stems from the exciton fine structure,^28^ the intervalley and the exciton–phonon coupling effects,^39^ and the spectral diffusion.^40^ Therefore, the record sharpness of the PbS NPLs studied here points to (i) an absence of spectral diffusion, (ii) a reduced exciton–phonon coupling and/or (iii) a different excitonic origin of the emission. Strikingly, we observe an additional discrete peak (arrow in Figure 2a) close to the ZPL on the PL spectra. A detailed analysis of the PL spectra around the ZPL (conducted on another NPL, Figure 2f) unveils that this discrete peak appears in both, the Stokes and anti-Stokes part of the PL spectra. Such a well-defined peak observed around 2.4 meV for most of the NPLs is most likely stemming from the thickness breathing mode (which would be 2.4 meV for a 1.6 nm thick PbS NPL, see Figures S7 for TEM images of PbS NPLs exhibiting the thickness of 1–2 nm).^41,42^ This feature, previously observed on PL spectra of individual CdSe^43^ and InP^44^ NCs at cryogenic temperatures, is the fingerprint of confined acoustic phonon modes at about 10 K, which corresponds to the base temperature in coldfinger cryostats for such sample preparation.

**Figure 2:**
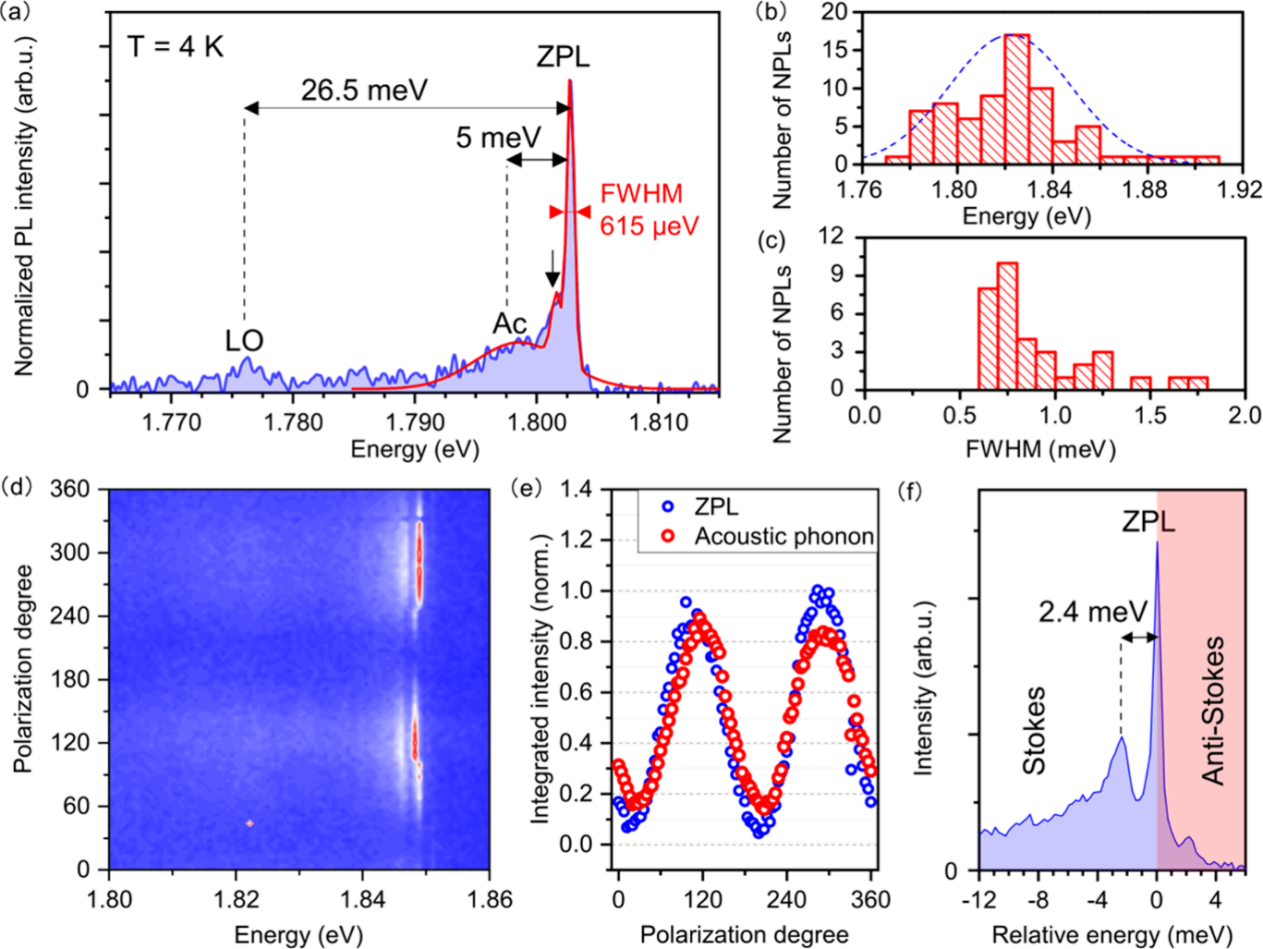
PL of single PbS NPLs at *T* = 4 K. (a) Micro-PL spectrum of a single PbS NPL (1s exposure time) featuring sub-meV emission and a red-shifted phonon sideband with LO-phonon replicas (LO) and acoustic phonon (Ac) contributions. (b) Distribution of the emission energy centered around 1.82 eV and (c) fwhm of the narrowband part of single PbS NPL emissions, obtained by measuring 71 individual NPLs. 74% of the measured emission lines exhibit sub-meV line widths. (d) Polarization-dependent PL measurement of a single PbS NPL and (e) the respective normalized PL intensity obtained in different spectral ranges as a function of the linear polarization angle of the emitted light. The blue dots represent the ZPL (δ = 0.90), the red dots correspond to the acoustic phonon sideband (δ = 0.71). (f) Normalized sum of the 91 PL spectra from the measurement in (d) and illustration of Stokes and anti-Stokes PL of the acoustic phonon sidebands of a single PbS NPL.

Notwithstanding unprecedented sharpness of their emission lines, PbS NPLs exhibit striking linear polarization properties (Figure 2d), which are analyzed by utilizing a rotating half-wave plate followed by a polarizer. In Figure 2e, integrated intensities of the ZPL and the acoustic phonon sideband for the various polarization angles are reported. From the angle dependent PL intensity, we evaluate the polarization degree, δ, as δ = (*I*_max_ – *I*_min_)/(*I*_max_ + *I*_min_) where *I*_max_ (*I*_min_) are the maximum (minimum) PL intensities. The ZPL displays a high polarization degree δ = 0.90 (for similar data from a second PbS NPL, we refer to Figure S8). The acoustic phonon sideband (from −1 meV to −8 meV in Figure 2f) shows a slightly lower polarization degree of δ = 0.71, while maintaining the same polarization angle as the ZPL.

The emission polarization of single NPLs is influenced by their aspect ratio,^31^ as well as the orientation of the NPL with respect to the substrate. A dipole orientation in the plane of the substrate will show a maximum polarization degree, whereas dipoles with orthogonal orientations are expected to appear as unpolarized emission (details in the SI, section B, Figure S8). The high degree of linear polarization in PbS NPLs indicates that the excitonic transition in individual PbS NPLs exhibits a polarization component, attributed to a linear 1D or 2D dipole. Furthermore, the alignment of the dipole is nearly ideal to the substrate plane (similar to observations shown in Figure 1). The aspect ratio is PbS NPLs is approximately 2, leading to anisotropic lateral electronic confinement and therefore contributing to the enhanced degree of observed polarization. It is important to note that the degree of polarization may depend on further factors that we do not study in detail here, such as selection rules of the allowed excitonic states and the respective oscillator strengths or possible effects of absorption polarization, by which the recombination of specific exciton types can be favored depending on the excitation energy and the energy spectrum of the NPL (further discussion in the SI, section C).

Figure 3 shows the comparison of a PbS NPL ensemble with single NPL PL to study the origin and excitonic nature of the emission in more detail. Figure 3a includes the temperature-dependent PL spectra of a PbS NPL ensemble (Figure S9 shows the data with an even more precise stepwise increase in temperature). We find an increasing slope of the high-energy PL edge with decreasing temperature, indicating an increased relative intensity and a decreased spectral width of the bandgap-associated emission. The PL spectrum of a single NPL at 4 K in Figure 3b, as well as the distribution of emission energies discussed in Figure. 2b, shows good overlap with the emission edge of the ensemble emission at *T* = 4 K in Figure 3a.

**Figure 3:**
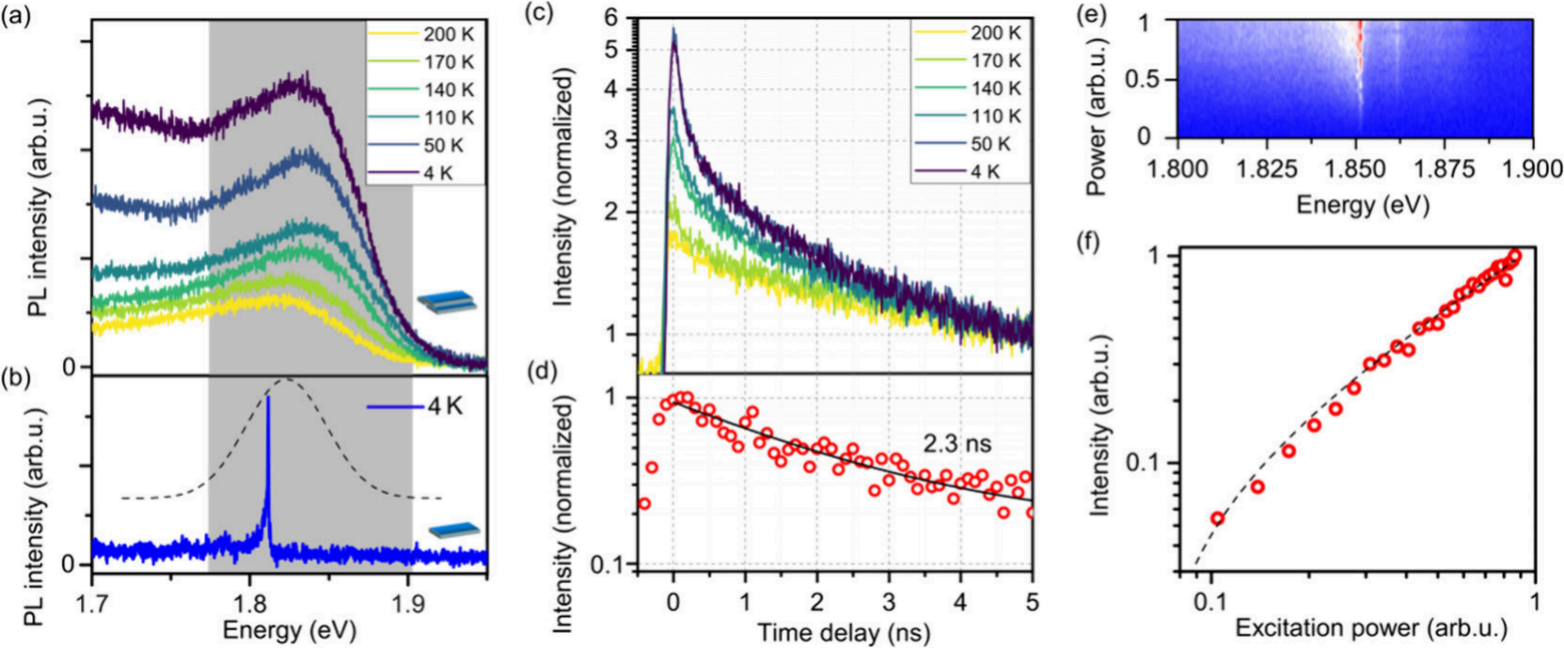
(a) Temperature-dependent PL spectra of an ensemble of PbS NPLs and (b) PL spectrum of a single PbS NPL at 4 K. The gray shaded area indicates the spectral range selected for lifetime measurements. The black dashed line represents the energy distribution of emissions from single PbS NPLs at 4 K in Figure 2b. (c) Fluorescence lifetime measurements of an ensemble of PbS NPLs, normalization is performed at a ‘long’ time scale (5 ns in this case) to emphasize the fast component and its strong temperature dependence. (d) Fluorescence lifetime measurement of a single PbS NPL. A monoexponential decay model is applied (solid, black line). (e) Excitation laser power dependent PL spectra of a single PbS NPL and (f) the respective normalized integrated PL intensity of the ZPL showing a linear increase with the excitation power.

The spectral region of the emission is then filtered (shaded gray area in Figure 3a, b) and detected with an avalanche photodiode to perform PL decay measurements for temperatures from 4 to 200 K (Figure 3c). The decay dynamics occur on two distinct time scales: a slow component (τ_1_ ≥ 6 ns) and a fast component (τ_2_ in the subnanosecond range), with detailed data provided in Figures S9. As the temperature increases to 160 K, we observe a lengthening of the fast decay component from 300 to 750 ps. The fast decay component, τ_2_, remains stable between 10 and 70 K, with most changes occurring above 70 K, which is consistent with the findings of Canneson et al.^45^ on trion emission in CsPbBr_3_ quantum dots. At temperatures of 170 K, the fast decay component disappears. This vanishing of the fast component could be explained by the thermal energy overcoming the binding energy of the excitonic complex, allowing us to estimate the trion binding energy to be ∼14.7 meV, which is close to the theoretical values reported in the literature for PbS NPLs of similar dimensions.^46^ (similar temperature-dependent results for PbS NPLs ensembles can be observed in Figure S10 and S11). These dynamics stand compared to the PL decay of PbS nanocrystals, which typically occurs on a microsecond time scale (see Figure S12 for PL lifetime measurement of ensemble PbS NPLs at RT).^47,48^ We attribute the presence of the fast component to the generation of higher order excitonic complexes (such as trions), additionally favored by a pile-up effect due to the high repetition rate of the pulsed laser of 82 MHz used for photoexcitation in the temperature-dependent measurements.

The PL decay of a single PbS NPL at 4 K is shown in Figure 3d, and data is best modeled with a monoexponential decay, even though it cannot be ruled out completely that a smaller fraction with a slower decay component is present also in this case. A lifetime of 2.3 ns is extracted, which is in the realm of lifetimes reported for CdSe^49^ and InP^50^ NCs.

A further tool to investigate the excitonic origin of NPLs and nanocrystals are excitation power dependent measurements.^51,52^ We perform these measurements under CW excitation with results shown in Figure 3e. A linear increase of the integrated PL intensity of the ZPL (Figure 3f) and the lack of additional spectral features with growing excitation power rule out multiexcitonic emissions as well as recombination processes that are independent of the excitation power (such as those involving trap or defect states).

By considering all observations up to this point, the question arises whether the narrow-band emission in single PbS NPLs can either be attributed to the presence of neutral excitons or trions, i.e. an exciton in the presence of an additional unpaired charge carrier. Owing to their specific spin structure,^53^ optical properties of trions (positive and negative) include rather temperature-independent emission characteristics.^54^ This results in trion emission having a rapid initial decay phase, which is indicative of swift radiative recombination. This phase is faster than the decay of dark excitons, yet slower than that of bright excitons.^54^ The unique dynamics of neutral excitons, which are susceptible to temperature due to the interplay between optically inactive (dark) and active (bright) states, are in stark contrast with the more temperature-stable behavior of trions. However, the proportion of trion transitions is sensitive to temperature, particularly in relation to their binding energy. When thermal energy surpasses the trion binding energy, the trion emission effectively vanishes. This aligns well with observations from the PbS NPL ensemble PL decay as a function of temperature, which demonstrates that, while the rate of fast PL decay remains constant, its relative weight diminishes with an increase in temperature. Based on these factors, we assume the fast decay of the narrowband emission of single PbS NPLs at 4 K is initiated by a trion transition. The sign of the trion does not affect the conclusion but further studies on the magneto-optical properties of the trion will aim at unveiling the sign of the trion and their spin dynamics.

To characterize the spectro-temporal dynamics in PbS NPLs, we analyze PL time traces of individual NPLs at a cryostat temperature of 4 K. These dynamics are key to understanding the influence of the nanomaterials’ surroundings on their photophysical properties. Figure 4a shows 300 consecutively recorded spectra with 1 s exposure time each, exhibiting a highly stable emission of PbS NPLs. All recorded spectra are summed up and normalized to obtain a time-integrated spectrum over 300 s (dashed red line in Figure 4b). A second approach addresses the spectral diffusion by rescaling the *x*-axis of each individual spectrum to match the emission energy of the strongest emission peak, followed by summing up these adjusted spectra and normalizing them (solid blue line in Figure 4b). The excellent overlap between the normalized sum of spectra and the spectral diffusion-compensated normalized sum of spectra underpins that the effect of spectral diffusion in the single PbS NPL emission is almost negligible over 5 min (see Figure S13 and S14 for more time trace measurements of individual PbS NPLs). The distribution of the central emission energy, derived from applying a Gaussian line shape to each spectrum, is presented in Figure 4c and reveals sub-meV (fwhm of 0.4 meV) spectral diffusion. The reduced fast spectral dynamics, which typically occur due to the Stark effect induced by trapped surface charges, points to a low density of surface traps. The application of CdCl_2_ in a postsynthetic step for surface passivation has been identified as an effective strategy for addressing dangling bonds via X- and Z-type binding to Pb^2+^ and S^2–^ surface sites, respectively, and contributes to a reduced trap state density.^25,26^ Midgap trap states are also expected to be of low level of significance due to the high crystallinity and well-balanced stoichiometry (Figure 1 and also Manteiga et al.^20^), as well as an expected robustness of PbS NPLs against off-stoichiometry predicted in *ab initio* simulations.^55^Figure 4d shows the time-dependent intensity of the emitted signal shown in Figure 4a, within the spectral region indicated with dashed lines. A stable emission is observed over long times with no clear traces of blinking and only slow, low-magnitude drifts of the intensity, which is yet another argument for a low trap-state density in PbS NPLs.

**Figure 4:**
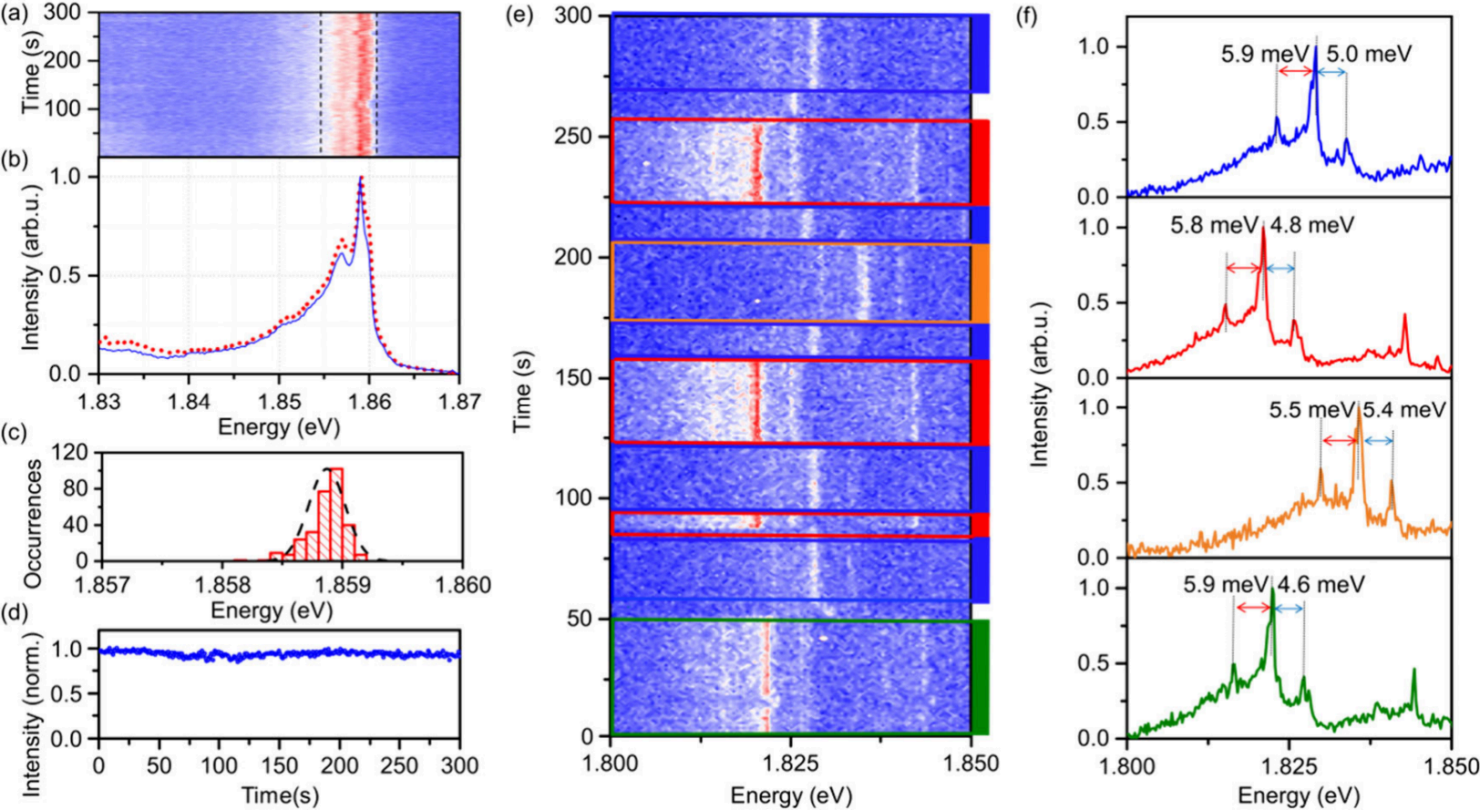
Spectral and temporal dynamics of single PbS NPLs. (a) PL time traces of a representative PbS NPL, exhibiting minimal spectral diffusion and blinking. (b) Normalized sum (red, dashed line) of the 300 PL spectra from (a). The blue line features the normalized sum of spectra which is corrected for spectral diffusion by rescaling the *x*-axis of each spectrum to the emission energy of the strongest emission peak. (c) Distribution of the central emission energy of the emission (obtained by fitting each spectrum in (a)) and (d) intensity time trace from the emission in (a) within the integration range shown by the dashed line. (e) PL time trace of a single PbS NPL, revealing combined spectral diffusion and blinking with discrete jumps. (f) Normalized sum of spectra for the four distinct emission states observed in (e), with the colors of the spectra corresponding to the selected ranges in (e).

While the low spectral diffusion is a common feature of the studied NPLs, the spectral characteristics can vary considerably. Figure 4e illustrates the spectral evolution of another PbS NPL, which displays several discrete spectral jumps over time. Four distinct spectral positions were identified, and for each of these we show the normalized sum of the respective spectra in Figure 4f by using the same color. Despite the differences in spectral position, the spectra are almost identical: The ZPL as well as observed Stokes and anti-Stokes PL features with phonon energies of around 5–6 meV do not change significantly. Another noteworthy feature is the lack of a memory effect^56^ at the spectral jumps, which indicates that the spectral positions correspond to discrete localized states that are not correlated or affected by the previous state of the NPL. These observations could be consistent with a trion emission experiencing four different Coulomb environments (e.g., four localized trapping sites) where the spectral positions are determined by the quantum-confined Stark effect induced by a charge carrier trapping. The absence of spectral diffusion (see Figure S15) at each spectral position indicates that the charges remain strongly localized as hopping charges would cause Stark effect fluctuations.

In conclusion, we have synthesized highly crystalline, ultrathin 2D PbS NPLs with CdCl_2_ ligands used for surface passivation. A comprehensive analysis of the PL properties of these PbS NPLs is conducted at cryogenic temperatures. The results reveal that single PbS NPLs exhibit strong and linearly polarized emission at 4 K, showcasing sub-meV line widths significantly narrower than those observed in spherical nanocrystals of similar materials. These findings highlight the unique optical properties conferred by the 2D geometry of the PbS NPLs. Time-resolved PL measurements confirm that the narrow emission originate from trions. The trion state in PbS NPLs demonstrates stable emission with minimal spectral diffusion and the absence of blinking over minutes. Additionally, PbS NPLs exhibit new spectral diffusion characteristics, which lack a memory effect. Our findings not only advance the fundamental understanding of colloidal 2D semiconductor NPLs but also emphasize their significant potential for advancing the next generation of optical and quantum technologies.

## Supporting Information Available

Brief description of the PbS NPLs synthesis as well as the polarization-dependent PL analysis; discussion of phonon sidebands in the PL spectra; TEM image, HR-HAADF-STEM and grazing-incidence wide-angle X-ray scattering diffraction of PbS NPLs; exemplary PL spectra of single PbS NPLs and localized emissions at 4 K; temperature-dependent PL spectra and photoluminescent lifetime measurements of PbS NPL ensembles; PL time traces, spectral diffusion and blinking behavior of individual PbS NPLs; fitting results of specific PL emissions of PbS NPL (PDF)
