# Supplementary material for: Sub-millielectronvolt Line Widths in Polarized Low-Temperature Photoluminescence of 2D PbS Nanoplatelets
Source: Nano Lett. 2024 Nov 22;24(51):16293–300. doi: 10.1021/acs.nanolett.4c04402 (PMC11674223; doi:10.1021/acs.nanolett.4c04402)
Supplement: Supplementary file 1 — nl4c04402_si_001.pdf [file nl4c04402_si_001.pdf]

Supporting Information

Sub-millielectronvolt Line Widths in Polarized Low-Temperature Photoluminescence of 2D PbS Nanoplatelets

Pengji Li[‡,1], *Leon Biesterfeld*[‡,2,3,4], *Lars F. Klepzig*[2,4], *Jingzhong Yang*[1], *Huu Thoai Ngo*[5], *Ahmed Addad*[6], *Tom N. Rakow*[1], *Ruolin Guan*[1], *Eddy P. Rugeramigabo*[1], *Ivan Zaluzhnyy*[7], *Frank Schreiber*[7], *Louis Biadala*[*,5], *Jannika Lauth*[*,2,3,4,8], *Michael Zopf*[*,1,8]

[1]Institute of Solid State Physics, Leibniz University Hannover, Appelstraße 2, D-30167 Hannover, Germany.

[2]Cluster of Excellence PhoenixD (Photonics, Optics, and Engineering – Innovation Across Disciplines), Welfengarten 1A, D-30167 Hannover, Germany.

[3]Institute of Physical and Theoretical Chemistry, Eberhard Karls University of Tübingen, Auf der Morgenstelle 18, D-72076 Tübingen, Germany.

[4]Institute of Physical Chemistry and Electrochemistry, Leibniz University Hannover, Callinstr. 3A, D-30167 Hannover, Germany.

[5]Université de Lille, CNRS, Centrale Lille, Université Polytechnique Hauts-de-France, Junia-ISEN, UMR 8520 - IEMN, F-59000 Lille, France.

[6]Université Lille, CNRS, INRAE, Centrale Lille, UMR 8207 − UMET- Unité Matériaux et Transformations, F-59000 Lille, France.

[7]Institute of Applied Physics, Eberhard Karls University of Tübingen, Auf der Morgenstelle 10, D-72076, Tübingen, Germany.

[8]Laboratory of Nano and Quantum Engineering, Leibniz University Hannover, Schneiderberg 39, D-30167 Hannover, Germany.

Corresponding Authors

*E-mail: michael.zopf@fkp.uni-hannover.de

*E-mail: jannika.lauth@uni-tuebingen.de

*E-mail: louis.biadala@iemn.fr

Supplementary Notes

A. Methods

Chemicals. Acetonitrile (⩾ 99.5 %), cadmium(II) chloride (99.99 %) isopropanol (⩾ 99.5 %), lead(II) oxide (⩾ 99.99 %), methanol (⩾ 99.8 %), *n*-octylamine (99 %), oleic acid (⩾ 90 %), rhodamine 6G (~95 %), triethylamine (⩾ 99 %), trifluoroacetic acid (99 %), and trifluoracetic anhydride (⩾ 99 %) were purchased from Sigma-Aldrich/Merck. n-Hexane (99.99 %) and thiourea (99 %) were purchased from Alfa Aesar. Ethanol (99.9 %) was purchased from Acros. Tetrachloroethylene (⩾ 99.9 %) was purchased from Merck-Millipore. The *n*-Octylamine and oleic acid were degassed *via* the freeze-pump-thaw method three times prior to being stored and used inside a nitrogen-filled glovebox. All other reagents were directly used as received from the listed suppliers without further purification.

All synthetic steps were performed under the inert gas conditions inside a nitrogen-filled glovebox, unless explicitly stated otherwise.

Preparation of the Lead Oleate Precursor. Lead oleate was synthesized *via* an established method described by Hendricks *et al.*[1] For the PbS NPL synthesis, lead oleate (365 mg, 0.47 mmol) was weighed into a 8 ml screw cap vial and dissolved in a mixture of *n*-hexane (2.3 ml), *n*-octylamine (1.5 ml), and oleic acid (0.8 ml) by stirring at 35 °C until complete dissolution.

Preparation of the Thiourea Precursor. The thiourea precursor was prepared by dissolving thiourea (180 mg, 2.36 mmol) in n-octylamine (4.5 ml) under continuous stirring at 35 °C. The mixture was stirred for at least 30 h before being used in the PbS NPL synthesis.

PbS NPL Synthesis. The PbS NPLs were synthesized following a procedure by Manteiga Vázquez *et al.*[2] The 8 ml screw cap vial containing the premixed lead oleate solution was allowed to heat up to 35 °C and 0.5 ml of the thiourea precursor solution were rapidly injected. After a reaction time of 20 minutes, the solution exhibited a bronze to dark-brown color and the PbS NPLs were passivated by adding a solution of $CdCl_2$ (2.5 ml, 0.1 M) in a mixture of *n*-octylamine and oleic acid (volume ratio of 9:1) and subsequent stirring for additional 10 min. The passivated PbS NPLs were stored at -25 °C in the fridge of a nitrogen-filled glovebox.

For preparing the samples for optical measurements at cryogenic temperatures the PbS NPLs were first transferred to toluene. Briefly, the NPLs were precipitated by drop-wise addition of a mixture of isopropanol and ethanol (3:1) until visible destabilization of the colloidal solution, centrifuged at 2500 rcf for 10 min, and reprecipitated in dry toluene.

The PLQY was determined using a relative method described by Würth *et al.*[3] using Rhodamin 6G in dry ethanol as a reference dye with a known quantum yield of 0.95. Thereby the PLQY is given by

$$PLQY = 0.95 \cdot \frac{A_{sample}}{A_{reference}} \cdot \frac{1 - 10^{-Abs_{reference}}}{1 - 10^{-Abs_{sample}}} \cdot \left(\frac{n_{TCE}}{n_{EtOH}}\right)^2, \quad (S1)$$

with the integral area A of the PL measurement curve, the absorbance Abs, and the refractive index n of the solvent.

Transmission electron microscopy. Overview TEM images were obtained using a FEI Tecnai G2 F20 transmission electron microscope equipped with a field emission gun operating at 200 kV. Samples for TEM analysis were prepared by drop-casting the colloidal PbS NPLs onto carbon-coated copper grids (300 mesh) acquired from Quantifoil.

High-angle annular dark-field scanning transmission electron microscopy. A FEI TitanThemis 300 microscope equipped with a probe aberration corrector, which is operated at 200 kV, was used to acquire (HR)STEM images. The probe size was set to 0.1 nm with a convergence semiangle of 22.5 mrad. The collection angle of the HAADF detector was in the range 80−150 mrad. The contrast in an HAADF image is proportional to Z $\approx 1.7-2$, meaning that the bright contrast indicates relatively heavy atomic composition.

Grazing-Incidence Wide-Angle X-ray Scattering. GIWAXS diffractions patterns were measured at the beamline P03 of the PETRA III synchrotron facility (DESY, Hamburg) at an incidence angle of $\alpha_i = 0.4°$. For this, samples were prepared by drop casting the colloidal PbS solutions onto silicon wafers (5 mm x 5 mm, p-typed doped with boron, <100> surface, purchased from Plano).

Optical measurements at cryogenic temperature: The optical measurements at cryogenic temperatures for the ensembles of PbS NPLs and the individual PbS NPLs were performed using a closed-cycle helium flow cryostat (Montana Instruments, Cryostation C2). The drop-casted PbS NPL samples were prepared the following way: 5% weight fraction of polystyrene were added to the PbS NPL solution in toluene, which was then centrifuged at 500 rpm for 2 min. The solution was then drop-casted onto a flat silicon substrate covered with a gold film, acting as mirror to increase the yield of collected PL from the sample. For obtaining the emission spectra of NPL ensembles and single NPLs, they were excited using continuous wave diode laser light at 532 nm (Thorlabs) which was focused onto the sample by an objective (Mitutoyo, M Plan Apo NIR 100x) with a numerical aperture of 0.7. The emitted PL signal was guided to a spectrometer (Spectroscopy & Imaging GmbH) with 300 l/mm grating and detected by a CCD (see figure S4). We calibrated the spectral transmission of our setup and spectrometer by guiding a broad-band white light signal (Thorlabs SLS202L) through the optical setup, including the spectrometer. The recorded spectrum and the original spectrum of the white light source were used to calibrate the total spectral efficiency of our setup and spectrometer. Different NPL ensemble emissions have been studied at different positions on the sample. For the time-resolved PL measurements, the PbS NPLs were excited by pulsed laser light at 445 nm for the ensemble of NPLs, and at 500 nm for the single NPLs.

The repetition rate of the pulsed laser was 82 MHz. The signal was collected by an avalanche photodiode. For the time-resolved PL measurements on ensemble PbS NPLs, the data is fitted bi-exponentially:

$$I(t) = I_0 + A_1 e^{\frac{-t}{\tau_1}} + A_2 e^{\frac{-t}{\tau_2}}, \text{(S2)}$$

With the background intensity I_0 and two amplitude components A_1 and A_2. These correspond to the intensities of the slow and fast decay components, with respective lifetimes τ_1 and τ_2, For the single PbS NPLs. the data is fitted single-exponentially:

$$I(t) = I_0 + A e^{\frac{-t}{\tau}}, \text{(S3)}$$

where I_0 represents the background intensity and A denotes the amplitude and τ is the derived lifetime.

For the polarization-dependent measurements, the degree of polarization (DOP) was determined by $\delta = (I_{max} - I_{min}) / (I_{max} + I_{min})$, where I_{max} and I_{min} are the fitted maximum and minimum PL intensities, respectively.

B. Polarization-dependent photoluminescence analysis.

In this section, we present the theoretical model for the simulation of the polarization degree dependent on the NPL orientation on the substrate. To determine the orientation (Θ,Φ) of an individual nano-emitter, we employ the theoretical framework proposed in

Refence [4]. Our simulated scenario is as follows. As shown in [4], the degree of linear polarization of the emission is defined as:

$$\delta(\Theta) = \frac{I_{max} - I_{min}}{I_{max} + I_{min}}, \text{ (S4)}$$

where I_{min} and I_{min} are the minimum and maximum intensity. For 1D dipoles,

$$I_{min} = A sin^2\Theta + B cos^2\Theta, \text{ (S5)}$$

$$I_{max} = C sin^2\Theta + I_{min} \text{ .(S6)}$$

And for 2D dipoles,

$$I_{min} = A + B + (A - B + C)cos^2\Theta, \text{ (S7)}$$

$$I_{max} = C sin^2\Theta + I_{min}. \text{ (S8)}$$

The constants A, B, and C can be determined analytically. Particularly the simple expression for δ in the limit of high numerical aperture ($\theta_{max} = \pi/2$) for a 1D and 2D dipoles are:

$$\delta_{high\,NA,1D}(\Theta) = \frac{7}{8} sin^2\Theta, \text{ (S9)}$$

$$\delta_{high\,NA,2D}(\Theta) = \frac{7}{16} sin^2\Theta, \text{ (S10)}$$

respectively. For both 1D and 2D dipoles the polarization degree δ depends on the out-of-plane Θ. Moreover, in the limit of low numerical aperture, δ for 1D and 2D dipoles[4] are:

$$\delta_{low\,NA,1D}(\Theta) = \frac{sin^2\Theta}{(1 - ((\theta_{\max}^2)/2)sin\text{^}2\Theta + ((\theta_{\max}^2)/2)}, \text{ (S11)}$$

$$\delta_{low\ NA,2D}(\Theta) = \frac{sin^2\Theta}{\left(\left((\theta^2_{\max})/2\right) - 1\right) sin^2\Theta + 2}, \quad (S12)$$

respectively. If we use low numerical aperture, i.e., NA = 0.7, In the case of $n_1 = 1.5$ (Polystyrene index), and $n_2 = 1$ (medium index), we can apply Eqn.(S12) to show δ change with Θ, with

$$\theta_{\max} = arc\ sin\frac{NA}{n_1}. \quad (S13)$$

The calculation result is presented in Figure S8.

C. Polarization Effects in Anisotropic PbS Nanoplatelets

In our study we investigate two-dimensional PbS NPLs. The properties of trions are often deduced from nanoycrystals where a cubic symmetry is assumed. For instance, in caesium lead halide perovskite nanocrystals it was discussed that in cubic nanocrystals (O_h point group), both electrons and holes possess Γ_6 symmetry and are two-fold degenerate.[5] As a result, the trion states formed are also of Γ_6 symmetry and similarly two-fold degenerate, with all allowed polarizations and equal dipole matrix elements along the x, y, and z axes.

However, PbS NPLs exhibit strong shape anisotropy. These NPLs are only 1–2 nm thick, but significantly larger in the other two dimensions (16.0 ± 1.6 nm x 9.2 ± 1.2 nm, in our case). This pronounced shape anisotropy breaks the cubic symmetry. With this reduced symmetry, stricter selection rules govern optical transitions, allowing transitions in certain directions while prohibiting them in others. As a result, the trion emission in PbS NPLs may become highly polarized. A finite degree of fluorescence polarization has also been observed in related systems such as PbS/CdS quantum dots.[6] Although these quantum dots are expected to show isotropic, unpolarized emission due to their symmetry, a slight linear degree of polarization is observed experimentally. Possible reasons lie in irregularities in

the shape of the quantum dots, anisotropy in the CdS shell, or defects at the core/shell interface, all of which break the symmetry and lead to polarized fluorescence signals. Another key factor contributing to polarization is the renormalization of the optical electric field within the NPLs, caused by the contrast of dielectric constants between the NPL and its environment. It leads to a significantly altered local electric field, as has been observed in CdSe/CdS NPLs.[7] For our measurements, the single PbS NPLs have been embedded in polystyrene. The resulting dielectric mismatch can increase the emission anisotropy, strengthening the in-plane dipole moment and contributing to the higher degree of PL polarization observed in our system.

On the other hand, experimental factors pose a slight overestimation to the observed polarization. For example, the use of a 0.7 NA objective lens, as well as the random orientations of the NPLs in the polystyrene matrix, may lead to deviations in the measured polarization. In future studies, advanced measurement techniques may probe the polarization in greater detail. These may include the use of a high-NA objective lens, specially designed substrates to control the orientation and tilt angle of the NPLs, and the application of magnetic fields to further explore the underlying mechanisms and selection rules. We anticipate that these approaches will help us gain a more comprehensive understanding of the polarization behavior in PbS NPLs.

D. Phonon sidebands in the photoluminescence spectra

All emissions that we observe feature sidebands which we attribute to phonon-assisted emission processes. Usually, a low energy tail of the PL is seen, which we attribute to the coupling with acoustic phonons. Coupling to distinct optical phonon related features seems to be not very prominent in the PbS NPLs. Such coupling is common in semiconductor

nanocrystals and is typically explained by a mixture of surface-related and intrinsic effects[8]. The presence of localized surface-charges induces a polarization in the nanocrystal and therefore enhances coupling to longitudinal (LO) phonons. Due to the high crystal and surface quality and passivation in our PbS NPLs leading to little spectral diffusion and the presence of a narrow emission, this type of coupling is expected to be strongly reduced. Intrinsic effects include a reduced overlap of electron and hole wavefunctions in the NPL leading to an increased polarization in the nanocrystal and therefore enhanced coupling to LO phonons. In the case of PbS NPLs, the wavefunction overlap is maximized in the direction of strongest confinement, and the quality of the orthogonally oriented surface is high with negligible surface reconstruction. In contrast, coupling to acoustic phonons (as seen in the spectrum) is expected via deformation potential and piezoelectric interactions. Until now, this suppression of optical phonon modes has not been observed in PbS nanostructures, most likely because of strong internal dipoles caused by asymmetry in the polar facet orientation and the structure truncation. Due to the high crystal and surface quality with negligible surface reconstruction (see Figure 1) as well as the applied passivation in the 2D PbS NPLs, a high wavefunction overlap in the direction of strongest confinement is expected, and thereby strongly reducing the NPL polarization and LO phonon coupling. The observed low-frequency acoustic modes (both in Stokes and anti-Stokes PL) are most likely stemming from the thickness breathing mode.[9,10] The phonon energies are in the range of a few meV with slight changes from NPL to NPL (see Figure

4 and SI), which is reasonable for confined acoustic phonons that are more sensitive to symmetry and size. The observed phonon energy range aligns well with transient absorption spectroscopy results in similar (slightly smaller) PbS NPLs.[2]

Supplementary Figures and tables

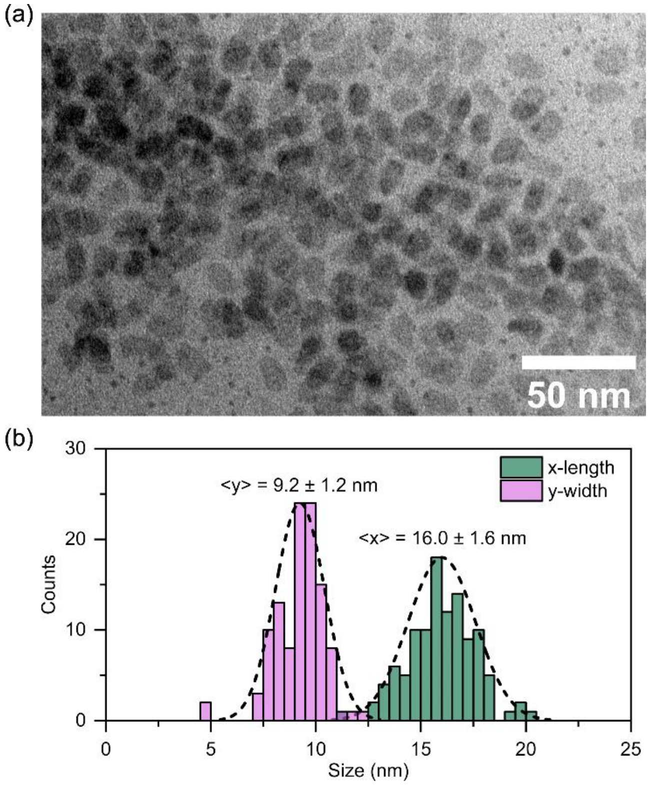

Figure S1. (a) Typical overview TEM image used for determining the lateral dimensions of the PbS NPLs. Within areas of higher concentration (compared to the overview image shown in the main manuscript), individual NPLs overlap with each other. (b) Corresponding size histogram, x-lengths correspond to the longest dimension of the NPLs, y-widths are the longest distance orthogonal to the x-length.

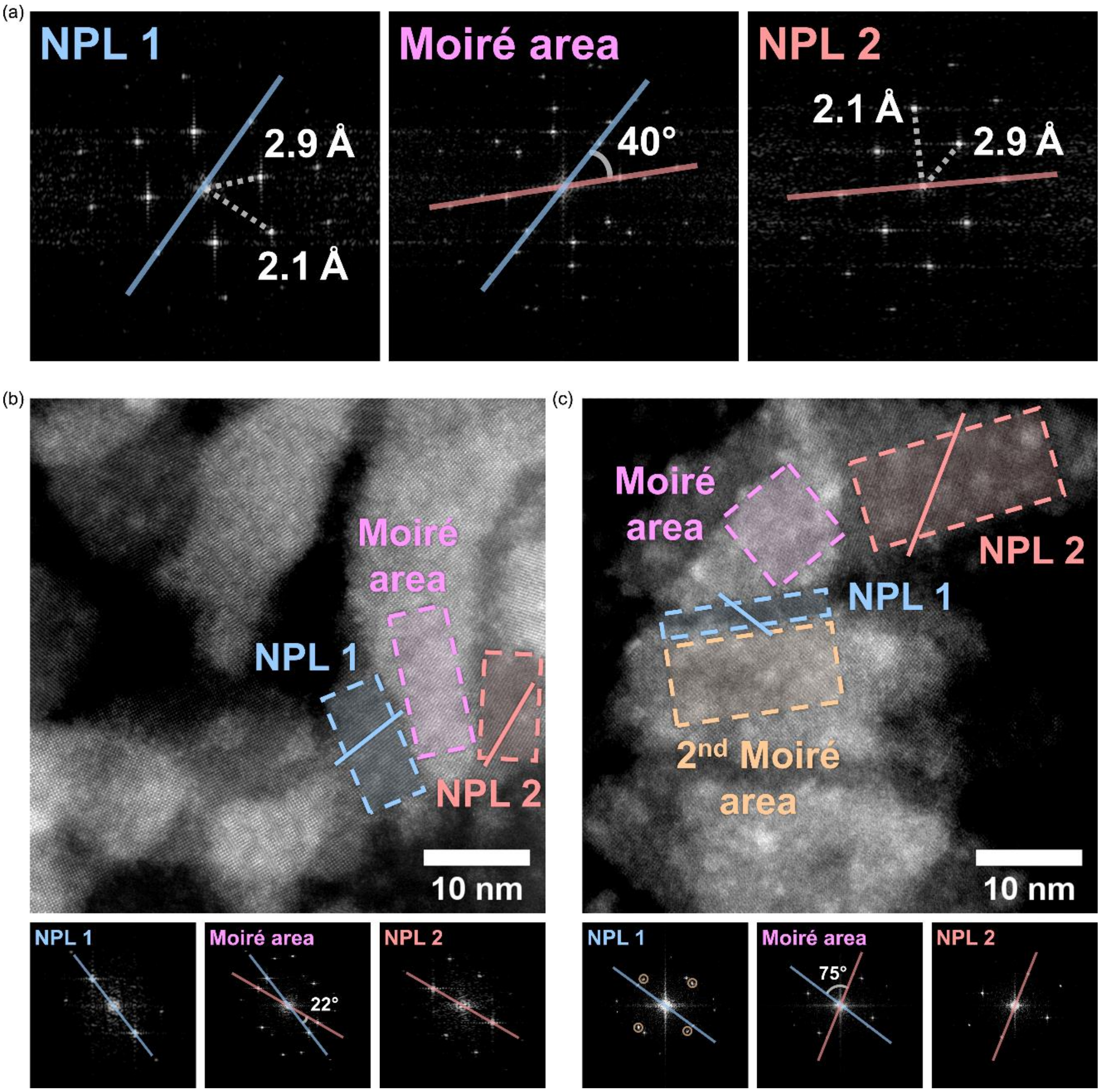

Figure S2. (a) FFT patterns corresponding to the two NPLs and the Moiré pattern depicted in Figure 1b of the main manuscript. The two PbS NPLs overlap with a twist angle of 40°. (b, c) HR-HAADF-STEM images and corresponding FFT patterns depicting additional examples of Moiré patterns formed by overlapping PbS NPLs with twist angles of (a) 22° and (b) 75°, respectively (compare to Figure 1b of the main manuscript).

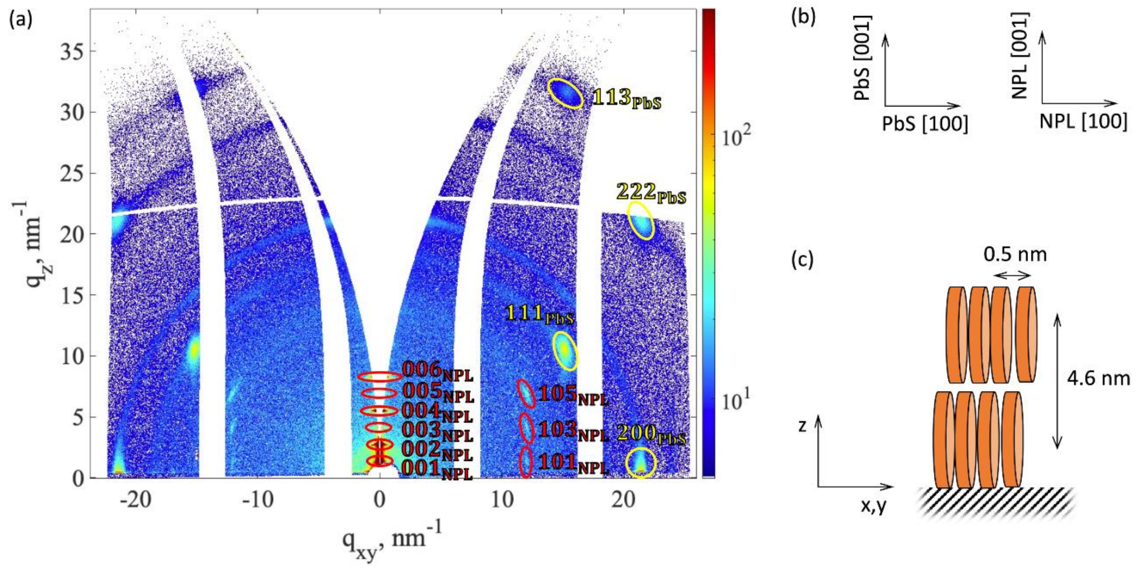

Figure S3. (a) Grazing-incidence wide-angle X-ray scattering diffraction pattern (background-corrected) of PbS NPLs drop-casted onto a silicon substrate (white areas are detector gaps). The presence of superlattice peaks (marked in red) at small values of the scattering vector q indicates the formation of a superlattice of the individual NPLs. At the same time, the Bragg peaks from the PbS atomic lattice (highlighted in yellow) allow us to confirm that the NPLs are oriented in a certain way within the formed superlattice. (b) Orientation of the crystallographic directions of the PbS atomic lattice and the superlattice of the NPLs. (c) Combining the Bragg peaks with the superlattice peaks, we propose the shown superlattice structure with the characteristic spacings of 0.5 nm and 4.6 nm between the individual NPLs in real space. Interestingly, the low intensity of the hkl peaks with $h + k + l = \text{odd}$ suggests, that the rows of NPLs might be shifted by half of a unit cell with respect to each other, resembling a body-centered superlattice. The small

distance of approximately 0.5 nm between the individual NPLs eliminates the possibility of relatively large organic ligands to fit between the NPLs.

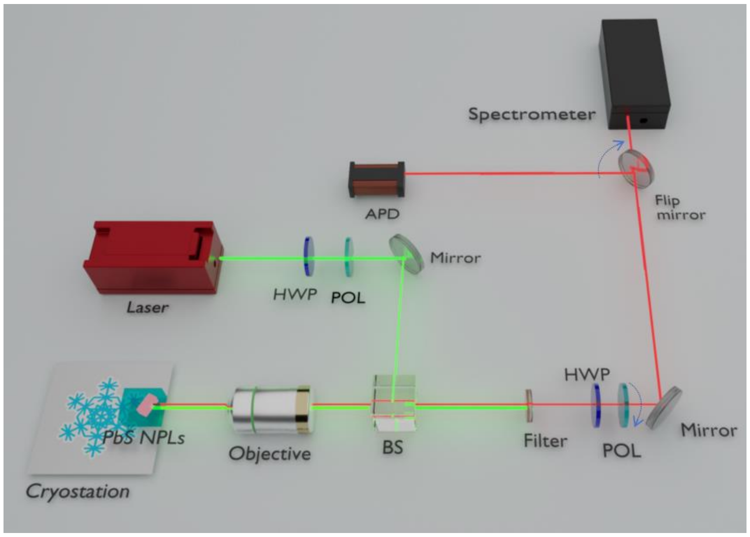

Figure S4: Sketch of the optical setup for micro-PL. BS: beam splitter. HWP: half wave plate. POL: polarizer. APD: Avalanche photodiode.

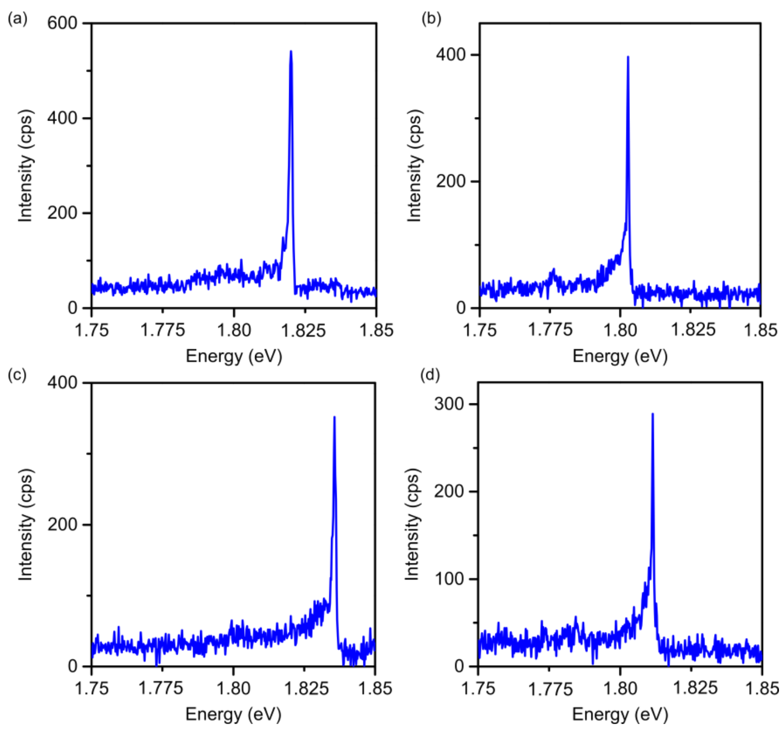

Figure S5: (a) – (d) Exemplary PL Spectra of four different single PbS NPLs at T = 4 K.

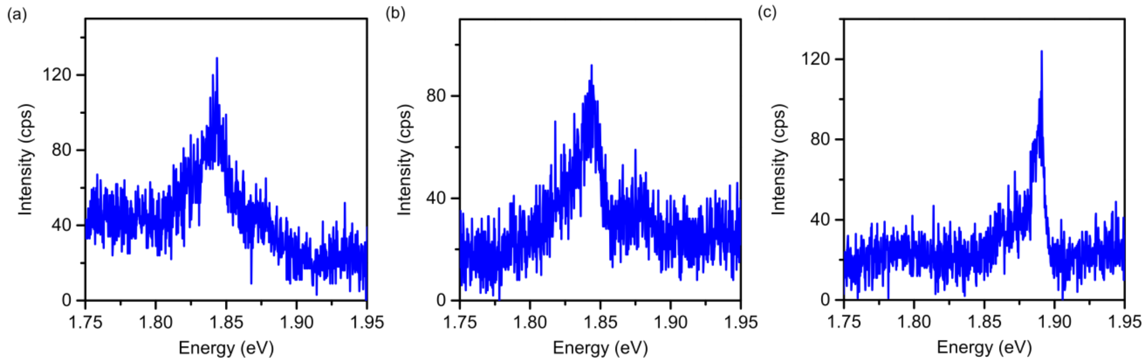

Figure S6: (a) – (c) Exemplary PL spectra (T = 4 K) of other localized emissions observed in the PbS NPL sample that do not exhibit narrowband emission.

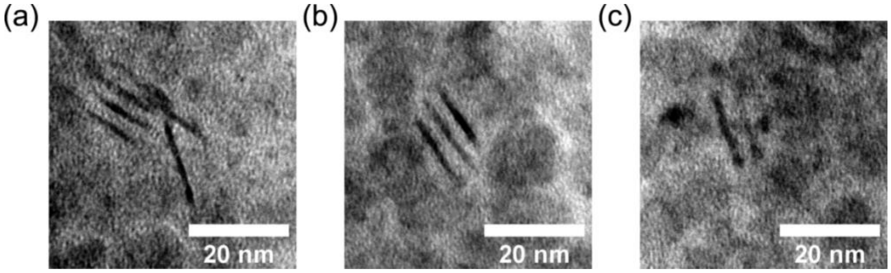

Figure S7: (a) – (c) Exemplary TEM images of PbS NPLs lying on their side exhibiting a thickness of 1-2nm.

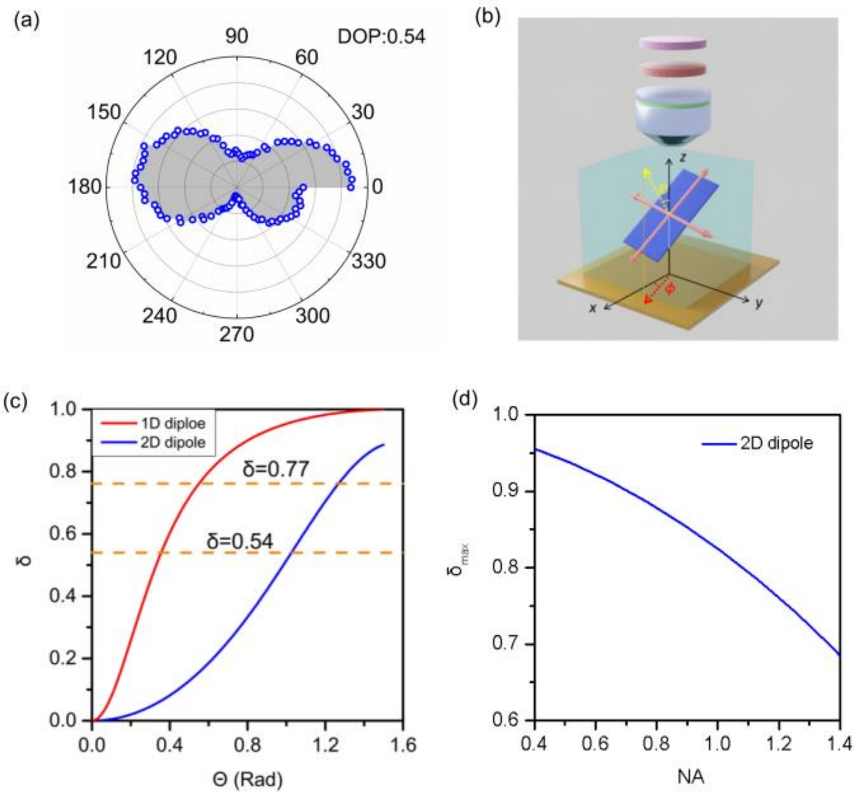

Figure S8: (a) Polarization angle dependent PL intensity of a single PbS NPL. The abrupt change observed between 0° and 360° is due to photobleaching during the measurement, resulting in a discontinuity in the PL intensity. (b) Sketch of the optical set-up and relevant

parameters of the model for estimating the polarization degree of a single PbS NPL. (c) Calculated polarization degree over the collection angle for an ideal 1D dipole (red) or 2D dipole (blue), assuming the experimental setup in (b). (d) Calculated maximum degree of polarization as a function of the numerical aperture of the objective, assuming the experimental setup in (b).

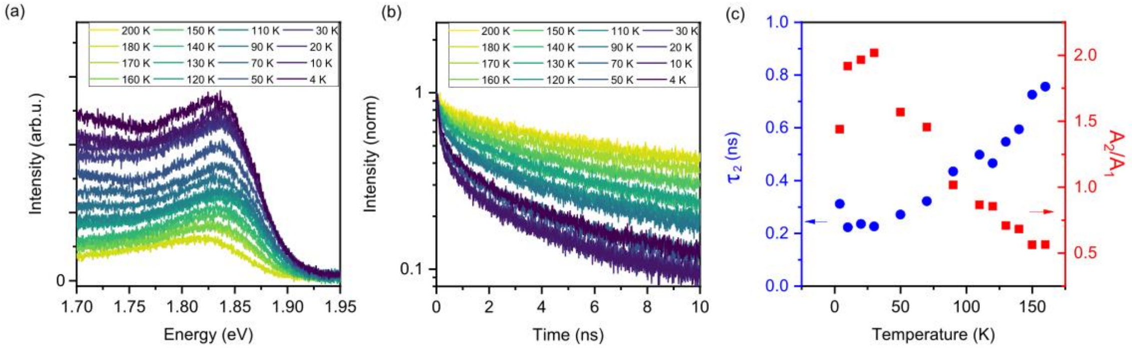

Figure S9. (a) Temperature-dependent PL spectra of the PbS NPL ensemble shown in Figure 3 of the main manuscript. (b) Fluorescence lifetime measurements of the ensemble of PbS NPLs. (c) Temperature-dependent decay time of the short component of the PL decay τ_2 (blue dot) and Temperature-dependent amplitude of short component of the PL decay over long component of the PL decay (red dot).

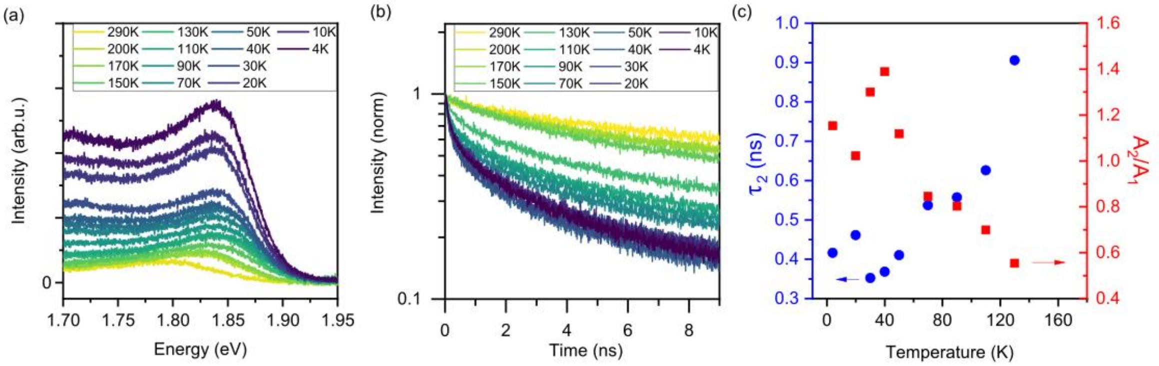

Figure S10. (a) Temperature-dependent PL spectra of a different ensemble of PbS NPLs. (b) Fluorescence lifetime measurements of the ensemble of PbS NPLs. (c) Temperature-dependent decay time of the short component of the PL decay τ_2 (blue dot), the short decay component τ_2 vanished at around 150 K and Temperature-dependent amplitude of short component of the PL decay over long component of the PL decay (red dot).

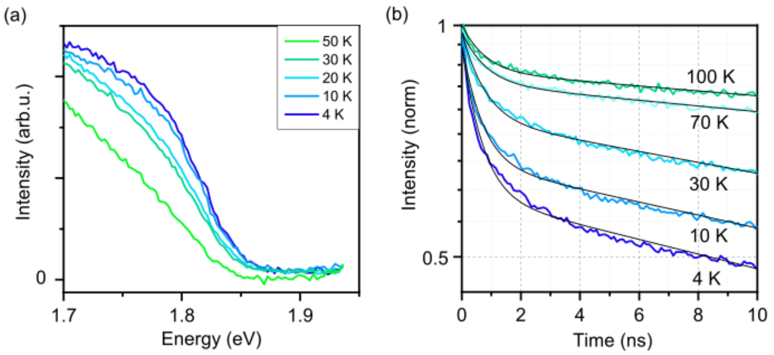

Figure S11. (a) Temperature-dependent, normalized PL spectra of a different ensemble of PbS NPLs. (b) Fluorescence lifetime measurements of the ensemble of PbS NPLs.

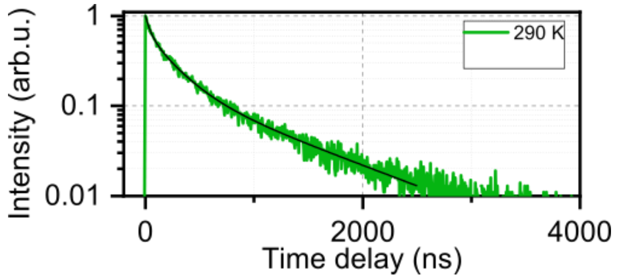

Figure S12: PL lifetime measurement of an PbS NPL ensemble at room temperature. A triple-exponential decay model is employed, with τ_1 = 42.6 ns, τ_2 = 231.3 ns and τ_3 = 956.2 ns.

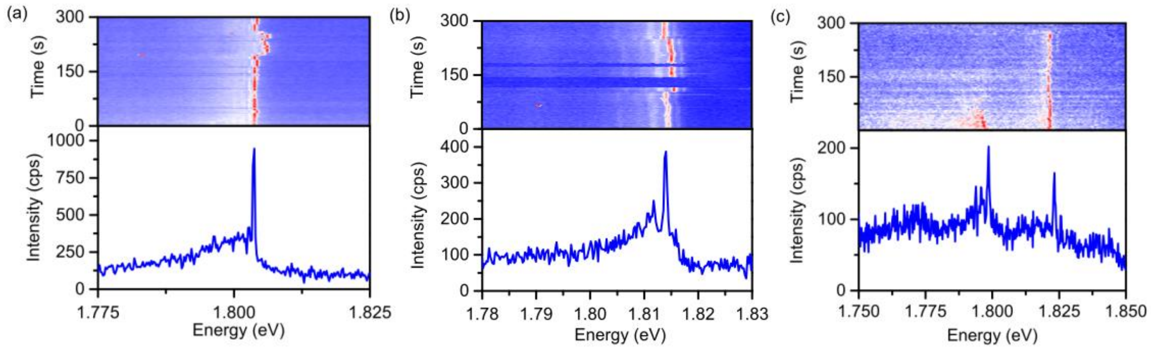

Figure S13: (a)-(c) PL time traces (top) and snapshot spectra (bottom) of three exemplary PbS NPLs exhibiting spectral diffusion.

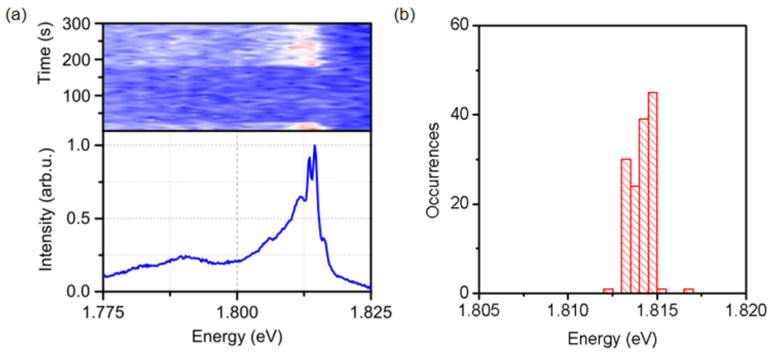

Figure S14: Only minor spectral diffusion and ultra-low blinking timescales in a representative PbS NPL. (a) The top part shows the PL time trace featuring low spectral diffusion and at the same time strong blinking behavior with a long off-time of around 150 s. The bottom part shows the normalized sum of spectra with the NPL in the bright state. (b) is the distribution of the central emission energy (obtained by fitting each spectrum in (a) with the NPL in its bright state).

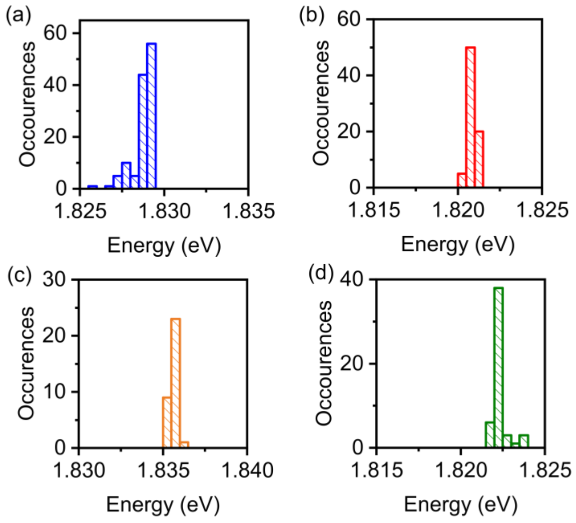

Figure S15: Distribution of the central emission energy of the strongest emission peak from four trion states in Figure 4e (obtained by fitting each spectrum in Figure 4e).

Table S1: The fitting results of specific PL emissions of PbS NPL derived from Figure 2a by using the Gaussian fittings.

	ZPL	Acoustic phonon
Position (eV)	1.8028	1.7986
FWHM (meV)	0.615	7.250

REFERENCES

(1) Hendricks, M. P.; Campos, M. P.; Cleveland, G. T.; Jen-La Plante, I.; Owen, J. S. A Tunable Library of Substituted Thiourea Precursors to Metal Sulfide Nanocrystals. *Science* **2015**, *348* (6240), 1226–1230.

(2) Manteiga Vázquez, F.; Yu, Q.; Klepzig, L. F.; Siebbeles, L. D. A.; Crisp, R. W.; Lauth, J. Probing Excitons in Ultrathin PbS Nanoplatelets with Enhanced Near-Infrared Emission. *J. Phys. Chem. Lett* **2021**, *12* (1), 680–685.

(3) Würth, C.; Grabolle, M.; Pauli, J.; Spieles, M.; Resch-Genger, U. Relative and Absolute Determination of Fluorescence Quantum Yields of Transparent Samples. *Nat Protoc* **2013**, *8* (8), 1535–1550.

(4) Lethiec, C.; Laverdant, J.; Vallon, H.; Javaux, C.; Dubertret, B.; Frigerio, J.-M.; Schwob, C.; Coolen, L.; Maître, A. Measurement of Three-Dimensional Dipole Orientation

of a Single Fluorescent Nanoemitter by Emission Polarization Analysis. *Phys. Rev. X* **2014**, *4* (2), 021037.

(5) Becker, M. A.; Vaxenburg, R.; Nedelcu, G.; Sercel, P. C.; Shabaev, A.; Mehl, M. J.; Michopoulos, J. G.; Lambrakos, S. G.; Bernstein, N.; Lyons, J. L.; Stöferle, T.; Mahrt, R. F.; Kovalenko, M. V.; Norris, D. J.; Rainò, G.; Efros, A. L. Bright Triplet Excitons in Caesium Lead Halide Perovskites. *Nature* **2018**, *553* (7687), 189–193.

(6) Hu, Z.; Kim, Y.; Krishnamurthy, S.; Avdeev, I. D.; Nestoklon, M. O.; Singh, A.; Malko, A. V.; Goupalov, S. V.; Hollingsworth, J. A.; Htoon, H. Intrinsic Exciton Photophysics of PbS Quantum Dots Revealed by Low-Temperature Single Nanocrystal Spectroscopy. *Nano Lett.* **2019**, *19* (12), 8519–8525.

(7) Ma, X.; Diroll, B. T.; Cho, W.; Fedin, I.; Schaller, R. D.; Talapin, D. V.; Wiederrecht, G. P. Anisotropic Photoluminescence from Isotropic Optical Transition Dipoles in Semiconductor Nanoplatelets. *Nano Lett.* **2018**, *18* (8), 4647–4652.

(8) Cui, J.; Beyler, A. P.; Coropceanu, I.; Cleary, L.; Avila, T. R.; Chen, Y.; Cordero, J. M.; Heathcote, S. L.; Harris, D. K.; Chen, O.; Cao, J.; Bawendi, M. G. Evolution of the Single-Nanocrystal Photoluminescence Linewidth with Size and Shell: Implications for Exciton–Phonon Coupling and the Optimization of Spectral Linewidths. *Nano Lett.* **2016**, *16* (1), 289–296.

(9) Goupalov, S. V. Low-Frequency Vibrations of Semiconductor Nanoplatelets. *J. Phys. Chem. C* **2019**, *123* (18), 11926–11932.

(10) Girard, A.; Saviot, L.; Pedetti, S.; Tessier, M. D.; Margueritat, J.; Gehan, H.; Mahler, B.; Dubertret, B.; Mermet, A. The Mass Load Effect on the Resonant Acoustic Frequencies of Colloidal Semiconductor Nanoplatelets. *Nanoscale* **2016**, *8* (27), 13251–13256.
